# Can Comprehensive Medical Reform Improve the Efficiency of Medical Resource Allocation? Evidence From China

**DOI:** 10.3389/ijph.2023.1606602

**Published:** 2023-12-21

**Authors:** Xiaoyang Sun, Liang Xu, K. M. Mehedi Adnan, Yusen Luo

**Affiliations:** ^1^ School of Finance and Economics, Jiangsu University, Zhenjiang, China; ^2^ Department of Agricultural Finance and Banking, Sylhet Agricultural University, Sylhet, Bangladesh; ^3^ School of Management, Jiangsu University, Zhenjiang, China

**Keywords:** China, pilot comprehensive medical reform, difference in differences, meta-frontier, efficiency of medical resource allocation

## Abstract

**Objectives:** To evaluate the impact of comprehensive medical reform on the efficiency of medical resource allocation in China.

**Methods:** This study employs the Slacks-Based Measure- Directional Distance Function (SBM-DDF) to estimate the efficiency of medical resource allocation (MRAE) in China, using panel data from 30 provinces during 2009–2021. Moreover, a multi-period Difference in differences (DID) model is developed to explore the effect of the comprehensive medical reform pilot (CMRP) strategy on efficiency of medical resource allocation in China.

**Results:** The results show that the average value of China’s medical resources allocation efficiency is 0.861 during the sample period. Coastal area has a higher MRAE than that in the inland area. The DID results show that the comprehensive medical reform pilot strategy has a good, long-lasting impact on the efficiency of medical resource allocation. And the results remain valid after a series of robustness analysis. Additionally, the comprehensive medical reform policy has heterogeneous impact on efficiency of medical resource allocation. The promotion effect is only statistically significant in the eastern and central regions, the groups of higher MRAE and larger population size.

**Conclusion:** China’s comprehensive medical reform policy can effectively promote the improvement of regional efficiency of medical resource allocation.

## Introduction

### Research and Propose

The lives of the Chinese people and those working in the medical and health fields are being threatened by the last COVID-19 epidemic. Over 767 million confirmed cases and over 6.9 million deaths have been reported globally until 2 July 2023 [1]. During the early stage of the COVID-19 epidemic, Wuhan was the most severely affected area in the world. However, lack of primary medical and health resources, lagging medical service capability, and other issues have severely hindered the effective implementation of prevention and control operations during epidemics [2]. The “weak link” in medical resources has been revealed as a result of this during time [3]. In order to save lives in future public health emergencies, even though COVID-19 is no longer considered a worldwide health emergency, it is imperative to figure out how to increase the efficiency of medical resource allocation. Additionally, it merits more research.

China has identified the key objectives for raising the quality and efficiency of medical treatment and medical resources in the nation’s 14th Five-Year Plan and Vision 2035, learning from the COVID-19 outbreak [4]. Statistics reported that government spending on health also increased from RMB481.626 billion in 2009 to RMB2071.85 billion in 2021, the average annual growth rate reaching 14.20 percent [5]. However, with the government’s support, it might also lead medical investment redundancy and decrease actual allocation capability of medical resources. China’s medical resources are uneven among its regions and between its urban and rural communities [6]. Moreover, the need to rationally distribute medical resources is growing. The ratio of medical resource output to input is known as the efficiency of medical resource allocation, and it can serve as a useful guide for hospitals and the government in order to maximize the distribution of medical resources. Based on this assumption, the primary goal of this study is to assess China’s efficiency in allocating medical resources. This will assist decision-makers in dynamically monitoring the efficiency of the allocation of medical resources. The prudent distribution of medical resources can be ensured by prompt response actions.

China’s authorities have enacted a number of medical and health policies, such as the new medical reform and the healthcare policy, to encourage the efficiency of medical resource allocation. However, there is disagreement regarding the following medical policies' real efficacy [3, 7–9]. Moreover, the difficulties of the medical reform strategy have gradually increased as it advances, and conflicts of interest among numerous departments cause frequent policy failures [10]. In view of this, In January 2015, State Council of P.R.C. selected four provinces, namely Jiangsu, Anhui, Fujian, and Qinghai provinces, as comprehensive medical reform as a pilot program to play the synergistic effect of various medical policies. In May 2016, the comprehensive medical reform pilot provinces were further enlarged by adding Shanghai, Zhejiang, Hunan, Chongqing, Sichuan, Shaanxi, and Ningxia into the second batch. However, whether the comprehensive medical reform pilot strategy is effective remains questionable. Thus, the second motivation of this study is to evaluate the policy effect of comprehensive medical reform pilot strategy from the perspectives of medical resource allocation efficiency is the core of this paper. This article is a panel study as far as contribution to public health is concerned. In contrast to longitudinal or single cross-sectional research, this article examines the evolution and changes within the same population over time. It is important to use statistical techniques to investigate potential factors influencing the efficiency of medical resource allocation. Therefore, another motivation for this research is to evaluate the advantages and disadvantages of the complete medical reform plan in real-world settings so that practical solutions to enhance MRAE can be provided.

According to our knowledge, this is the first study to examine the impact of a complete medical reform pilot strategy on the efficiency of resource allocation in medicine, which may provide some insight into how China and other developing countries might improve their capacity for resource allocation in medicine. The following are some potential innovations. First off, the technical level of the DMUs is assumed to be homogeneous in the available research when evaluating the efficiency of allocating medical resources. However, heterogeneity may exist if the disparity in production technique due to resource endowment and economic structure in each place [11]. In this study, we adopt the SBM-DDF model under meta-frontier to measure China’s efficiency of medical resource allocation, which can better capture the characteristics of medical resources allocation capacity. Secondly, there is little literature on the evaluation of the policy impact of comprehensive medical reform pilot policy. In this study, we adopt a multi-period DID model to assess the effectiveness of comprehensive medical reform pilot strategy from the perspectives of efficiency of medical resource allocation. It can lessen the endogeneity issue and improve the accuracy of estimation. Lastly, this study undertakes heterogeneity analysis from the perspectives of geographic location, efficiency of medical resource allocation, and population size. It is of great practical significance to give guidance for the government in implementing targeted medical reform policies.

### Institutional Background

The general quality of medical and health services has greatly improved since the new medical and health system reform was put into place in 2009. Still, there were a few key problems. The lack of a connection between health insurance, healthcare, and medicine slowed down the pace of change [8]. As a result, two batches of comprehensive medical reform including eleven pilot provinces have been implemented by China’s top medical reform group [12]. Pilot provinces must apply the partial first and single breakthrough theory in order to address the innate issues that exist. It is necessary to build a single management system for prescription drugs, medical insurance, and healthcare. The objectives are to safeguard the public’s interests and pique the motivation of medical professionals.

Several initiatives have been put in place by medical reform provinces to increase the efficiency of the allocation of medical resources. Anhui Province, for instance, abolishes sales commissions and markups on expensive medical supplies at public hospitals. The novel applications of “cloud film” and “medical after-payment” are promoted by Zhejiang Province. The province of Ningxia has established a telemedicine service system. Drug sales at state hospitals are now zero-profit in Jiangsu Province. The National Health Commission’s 2020 report [13] states that China’s experimental medical reform program has produced impressive outcomes in terms of systematization, integrity, and coordination.

### Literature Review

#### Efficiency of Medical Resource Allocation

Medical resources are the production variables that are used to deliver medical services. These factors typically include persons, medical costs, medical institutions, medical beds, medical facilities, equipment, knowledge, skills, and information [6]. In recent years, scholars have attempted to evaluate the efficiency of medical institutions or medical resources allocation [14]. To determine how well medical resources are allocated, there are essentially two approaches utilized, one of which is based on stochastic frontier analysis (SFA). SFA is an efficiency estimation method using the stochastic frontier production function. The model makes an estimate of the technical efficiency of DMUs by decomposing the error term [15]. Examples comprise: The stochastic frontier production function approach was used by Sheng et al. [16] and Thanassoulis et al. [17] to measure and compare the effectiveness of medical and health services in Chinese provinces and cities. The SFA model was used to evaluate the effectiveness of the German healthcare system. Frogner et al. [18] used SFA techniques to revisit the WHO efficiency evaluation of health systems in industrialized countries. The study found that the efficiency of medical resource allocation in the United States is greater than that evaluated by the WHO. The alternative method uses a non-parametric strategy called data envelopment analysis (DEA). DEA is a linear programming model expressed as the ratio of output to input [19]. For instance, Zheng et al. [20] used the SBM-DEA model to analyze the effectiveness of China’s rural medical service system from 2013 to 2017. Through the use of the SBM model, Zhao and Chen [21] also evaluated and contrasted the efficiency of medical and health expenditures in China’s urban and rural areas. Feng et al. [14] adopted a three-stage DEA model to evaluate medical and health resources allocation efficiency. Gong et al. [22] measured the resource allocation of medical and health financial expenditure in Chinese cities by DEA technology.

#### Medical Reform Policy

Numerous scholars have been interested in the execution of the medical reform program and have evaluated its implications on policy from a range of perspectives. For instance, Shu et al. [23] argued that the implementation of the new medical reform boosted the accessibility of core medical services. According to Sun et al. [24], the new medical reform policy has boosted patient happiness, lowered the cost of vital pharmaceuticals, and raised the bar for basic medical security. The reform prioritizes health, strikes a balance between efficiency and equity [25–27]. He and Zhou [28] found that the new medical reform fell short of resolving the issue of “expensive medical treatment” on the basis of CHARLS data. Refer to the study of Liu et al. [29], doctors’ profit-seeking behavior raises the cost of medical care for patients, leading to the failure of the new medical reform program. This conclusion is consistent with their findings [29]. The objectives of the new medical reform during the 12th Five-Year Plan era were not met, Cheng et al. [8] added in another point. Lee et al. [30] utilized a logistic model to empirically examine the effect of medical reform on the standard of social services for the aged in China before and after the health reform in 2009. According to Tang et al.’s [31] empirical investigation, residents’ perceptions of their medical level, their happiness with medical care, and the cost of treatments were all affected by comprehensive pricing reform. The pilot comprehensive medical reform’s efficacy in reducing the problem of “expensive medical treatment” was proven by Nie and Feng [32] utilizing the DID approach.

In a nutshell even though there has been a wealth of recent research on the evaluation of medical resource allocation efficiency, there is still room for improvement. In this work, we empirically investigate how comprehensive medical reform affects the effectiveness of medical resource allocation, which can contribute to pertinent research on the influence of comprehensive medical reform on policy.

## Methods

### MRAE Measurement

#### SBM-DDF Model

The present literature primarily uses the conventional Banker-Charnes-Cooper (BCC) model [14, 33] or the Slacks-Based Measure (SBM) model [34] to calculate the allocation efficiency of China’s medical resources. Considering the heterogeneity of the regional production technology, the evaluation results of original DEA model, with the default assumption that all Decision-Making Units (DMUs) are homogeneous, might be inaccurate. Hence, following the study of Luo et al. [35], this study utilizes meta-frontier technology to calculate China’s medical resource allocation efficiency. Moreover, we adopt the Slacks-Based Measure- Directional Distance Function (SBM-DDF) model put forth by Fukuyama and Weber [36] to evaluate the efficiency score. The benefits of the SBM and DDF models are combined in the SBM-DDF model. This approach allows for the intended and undesirable outputs to change in different amounts while accounting for the slack variables of input and output [36]. It is often used in research on assessing efficiency. For instance, Sun et al. [37] and Qiu et al. [38]. So, in order to quantify the allocation efficiency of medical resources in China, we employ the SBM-DDF model under meta-frontiers as an evaluation technique. The specific SBM-DDF model is shown in the Supplementary File S10.

#### Input-Output Variables

The input indicators utilized in this study to assess China’s medical resource allocation efficiency include HE (Health Expenditures), HP (Health Personnel), MIB (Medical Institution Beds), and MI (Medical Institution). These indicators are based on the work of Feng et al. [14], Bilsel et al. [39], and Robinson et al. [40]. Outpatient Visit (OV), Discharge Rate (DR), and Bed Utilization (BU) are the components of the desirable output indicator. In contrast to earlier research, this one additionally selects MOR (Mortality in Observation Room) and EM (Emergency Mortality) as undesired output metrics. Correlation and descriptive analysis of input-output variables can be seen in Supplementary File S1.

### Econometric Model

#### Differences in Differences (DID) Method

Card and Krueger [41] proposed the applicability of DID theory to economics. Research objects must be separated into two groups in order to use the DID approach. There are two groups: the experimental group and the control group. Natural control experiments were constructed. The DID method allows us to determine whether there is a substantial difference between the two groups. Hence, the DID method is widely used to evaluate policy causal effects [41]. The Chinese government released two batches of pilot provinces in 2015 and 2016, respectively. In this study, we employ the multi-stage DID approach, which is based on the approach of Beck et al. [42] and Meyer [43], to calculate the net impact of the comprehensive medical reform strategy on China’s medical resource allocation efficiency. The following describes the benchmark model developed in this paper.

$$MRAE_{it}=\alpha_{0}+\alpha_{1}du*dt+\beta X_{it}+\mu_{i}+v_{t}+\varepsilon_{it}$$ (1)

Here, the policy impact of China’s pilot comprehensive medical reform is measured by du*dt, where *MRAE*_*it*_ is the study’s explanatory variable. *du* stands for the province’s dummy variable for comprehensive medical reform. When *du* = 1, it denotes the pilot area of the comprehensive medical reform; dt is the dummy variable of the pilot comprehensive medical reform’s implementation time. The control variable in this study is *X*_*it*_. Additionally, *ε*_*it*_ is the random disturbance term, and *μ*_*i*_ and *v*_*t*_ stand for individual fixed effects and time fixed effects, respectively.

The basic assumption of the multi-stage DID approach is that the selection of the treatment group is out of endogeneity. In other words, the treatment group and the control group share the identical change trend in the allocation efficiency of medical resources in each province before the policy shock of the comprehensive medical reform pilot strategy. According to the research of Liu et al. [44] and Li et al. [45], the event analysis method is further used in this paper to assess the parallel trends and dynamic effects between the treatment group and the control group. The particular model is depicted in Formula (2):

$$MRAE_{it}=\alpha_{0}+\alpha_{i}\sum_{i=1}^{6}du*{before}_{i}+\beta_{i}\sum_{i=1}^{6}du*after_{i}+\lambda X_{it}+\mu_{i}+v_{t}+\varepsilon_{it}$$ (2)

Where, the estimated coefficient *α*_*i*_ measures the parallel trend between the treatment group and the control group. When *α*_*i*_ is not significant, it indicates that before the implementation of the pilot policy of comprehensive medical reform, the MRAE in the pilot provinces and non-pilot provinces has a similar trend. The estimated coefficient *β*_*i*_ measures the dynamic effect of the pilot policy.

### Variable Selection

#### Dependent Variable (*MRAE*_*it*_)

The *MRAE* of Chinese provinces was calculated by the SBM-DDF model mentioned in Supplementary File S10.

#### Key Independent Variable (du*dt)

The core independent variable in this study is the interaction term du*dt. If province *i* belongs to the treatment group and year *t* is after the start of the pilot reform, du*dt equals 1, otherwise it equals 0.

#### Control Variables (*X*_*it*_)

To control the impacts of the other variables on *MRAE*, this study selects the following variables from the existing literature [34, 46]. The per gross domestic product (*lnPGDP*_*it*_) is defined as the log of the ratio of gross domestic product to population. The population (*lnPOP*_*it*_) is expressed as the logarithm of local population at the end of the year. Government health expenditure intensity (*Gov*_*it*_) is measured by the ratio of government health expenditure to government financial expenditure. The upgrading of industrial structure (*Idu*_*it*_) is represented by the ratio of the tertiary industry to the secondary industry. The innovation level (*lnPat*_*it*_) represented by the logarithm of the number of invention patent applications.

### Data Source and Variable Description

Based on the data availability and continuity, Hong Kong, Macaw, Taiwan and Tibet were not included in this research sample. Thus, this study utilizes a panel data of 30 Chinese provinces from 2009 to 2021. *China Statistical Yearbook* and *China Health Statistical Yearbook* provide the majority of the data for the relevant factors used in this paper. Table 1 displays the descriptive statistical analysis of the primary variables.

**TABLE 1:** Descriptive analysis. (China, 2009–2021).

Variable	Total sample			Treatment group			Control group		
	Obs.	Mean	Std. Dev.	Obs.	Mean	Std. Dev.	Obs.	Mean	Std. Dev.
MRAE	390	0.86	0.18	143	0.92	0.13	247	0.83	0.19
du*dt	390	0.18	0.38	143	0.49	0.50	247	0.00	0.00
lnPGDP	390	10.73	0.51	143	10.87	0.52	247	10.65	0.49
lnPOP	390	8.20	0.74	143	8.13	0.89	247	8.24	0.65
Gov	390	0.08	0.02	143	0.08	0.01	247	0.08	0.02
Idu	390	1.19	0.69	143	1.06	0.42	247	1.27	0.79
lnPat	390	9.40	1.48	143	9.71	1.67	247	9.22	1.33

Additionally, this study also draws the kernel distribution of MRAE level during 2009–2021 (see Supplementary File S2). The efficiency of China’s medical resource allocation has been seen to be left-skewed, and between 2009 and 2021, the kernel density peak shifts from the left to the right. The height of the peak rises as its width decreases. This is consistent with China’s present trend of improving the use of medical and health resources. Regional inequalities in the efficiency of medical allocations of resources are vanishing, exhibiting the characteristics of dynamic convergence. It has been noted that China’s medical resource allocation efficiency is left-skewed, and between 2009 and 2021, the kernel density peak moves from the left to the right. The peak’s width narrows while the peak height increases. This is in line with China’s current trend of enhancing medical and health resource efficiency. Regional differences in the allocation efficiency of medical resources are decreasing, displaying the traits of dynamic convergence.

## Results

### Characteristics of Province MRAE

The SBM-DDF model is used in this study to calculate the allocation efficiency of China’s medical resources from 2009 to 2021 under group frontier and meta-frontier. Supplementary File S3 illustrates the changing trend of that efficiency value. Based on the group frontier measure, it can be seen that China’s medical resource allocation efficiency has not changed significantly overall during the observation period. The efficiency value calculated under meta-frontier exhibits a trend toward decline from 2009 to 2011, followed by a period of relatively stable performance from 2012 to 2015. After 2016, the allocation efficiency of China’s medical resources has increased from 0.859 to 0.869, which indicates that the pilot policy of comprehensive medical reform has improved China’s MRAE to a certain extent.

Additionally, based on the group frontier measure, the allocation efficiency of medical resources is much larger than that based on the meta-frontier measure. It demonstrates that the conclusions drawn from the group frontier measure overstate the actual allocation capability of regional medical resource. The difference between the meta-frontier’s and group frontier’s technological levels is reflected in the TGR index. The overall changing trend of the TGR index from 2009 to 2021 is compatible with the value for efficiency of medical resource allocation under meta-frontier. The TGR score has been trending upward since 2019, which suggests that China’s medical and healthcare system has gradually advanced in terms of technological proficiency.

Table 2 shows the average level of efficiency of medical resource allocation for each region between 2009 and 2021. At the provincial level, the efficiency value in Tianjin, Shanghai, Zhejiang, Fujian, Guangdong, Hainan, Qinghai and Ningxia all equal to 1, reaching the production frontier. The mean value of MRAE in Liaoning, on the other hand, is the lowest among the findings of efficiency measurement under group frontier. However, Heilongjiang has the lowest efficiency rating under meta-frontier. Meanwhile, the TGR index in Heilongjiang is the lowest, at only 0.474. It demonstrates that Heilongjiang’s actual efficiency of medical resource allocation is only 47.4% of its potential efficiency, leaving 53.6% of space for improvement.

**TABLE 2:** Measurement results of medical and health resource efficiency in China. (China, 2009–2021).

East China				Central China				West China			
Province	Meta	Group	TGR	Province	Meta	Group	TGR	Province	Provinces	Group	TGR
Beijing	0.995	0.995	1.000	Shanxi	0.620	0.967	0.641	Inner mongolia	0.570	0.686	0.831
Tianjin	1.000	1.000	1.000	Jilin	0.581	1.000	0.581	Guangxi	0.987	1.000	0.987
Hebei	0.880	0.899	0.980	Heilongjiang	0.465	0.980	0.474	Chongqing	0.950	1.000	0.950
Liaoning	0.572	0.593	0.964	Anhui	0.913	1.000	0.913	Sichuan	0.908	1.000	0.908
Shanghai	1.000	1.000	1.000	Jiangxi	0.993	1.000	0.993	Guizhou	0.883	1.000	0.883
Jiangsu	0.987	0.989	0.998	Henan	0.994	1.000	0.994	Yunnan	0.934	1.000	0.934
Zhejiang	1.000	1.000	1.000	Hubei	0.874	0.990	0.881	Shaanxi	0.615	0.748	0.824
Fujian	1.000	1.000	1.000	Hunan	0.773	0.948	0.821	Gansu	0.892	0.969	0.921
Shandong	0.873	0.902	0.968					Qinghai	1.000	1.000	1.000
Guangdong	1.000	1.000	1.000					Ningxia	1.000	1.000	1.000
Hainan	1.000	1.000	1.000					Xinjiang	0.578	0.688	0.841
Average	0.937	0.943	0.993	Average	0.776	0.986	0.788	Average	0.847	0.917	0.923

At the regional level, East China has the largest efficiency of medical resource allocation, with the value of 0.937, which is much greater than Central China (0.776) and West China (0.847). The technology gap ratio in the eastern provinces is on average 0.993, which is significantly larger than the central region’s TGR of 0.788 and the western region’s TGR of 0.923. It shows that the actual production technology in East China is closest to that under meta-frontier, followed by West China.

**FIGURE 1:**
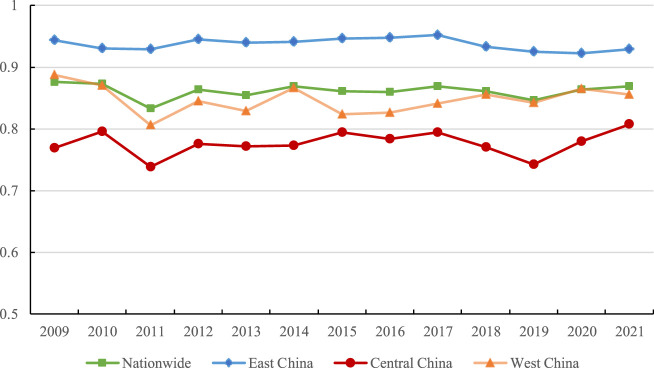
Trends of the efficiency of medical resource allocation in different regions of China from 2009 to 2021. (China, 2009–2021).

Moreover, this study also explores the changing trend of the allocation efficiency of medical resources in different regions of China (see Figure 1). It is clear that East China has the largest MRAE during the sample period, followed by West China and Central China. The efficiency value in East China ranges about 0.937 from 2009 to 2021, sustaining a high level of medical resource allocation capability. The allocation efficiency of medical resources in Central China increased from 0.769 in 2009 to 0.807 in 2021, at a growth rate of 0.406%. West China experienced a decreasing trend from 2009 to 2011, then exhibited an upward tendency from 2011 to 2021.

### Multicollinearity Analysis

The results of the multicollinearity test are shown in Supplementary File S4, with particular emphasis on the Variance Inflation Index (VIF). It can be found that VIF values of all the selected variables are below 10, with the highest value being 7.60. This shows that the variable data does not have a serious multicollinearity problem. Furthermore, at a 1% level of significance, the correlation coefficient shows a positive association between comprehensive medical reform and the allocation efficiency of medical resources.

### Benchmark Analysis

Table 3 reports the benchmark regression results of the pilot comprehensive medical reform policy on the allocation efficiency of medical resources in China. It can be found that the estimated coefficient of the core explanatory variable du*dt is significantly positive at a 5% confidence level. and the results remain valid after adding control variables and controlling industrial as well as year effect. This demonstrates that the comprehensive medical reform pilot program’s implementation can greatly boost all of medical resources. The pilot provinces' special attention to hierarchical diagnosis and treatment and their reinforcement of the management of contemporary hospitals may be the cause of the effectiveness of comprehensive medical reform.

**TABLE 3:** Benchmark analysis. (China, 2009–2021).

	(1)	(2)	(3)	(4)	(5)	(6)
du*dt	0.0889***	0.0173**	0.113***	0.0261**	0.0298***	0.0306***
	(0.0230)	(0.0085)	(0.0261)	(0.0103)	(0.0101)	(0.0108)
lnPGDP_it_					−0.0178	−0.0131
					(0.0219)	(0.0448)
lnPOP_it_					−0.0940	−0.0788
					(0.0844)	(0.0840)
Gov_it_					−0.3990	−0.9670**
					(0.4280)	(0.4880)
Idu_it_					0.0094	0.0050
					(0.0122)	(0.0173)
lnPat_it_					0.0042	0.0042
					(0.0091)	(0.0110)
Constant	0.845***	0.858***	0.876***	0.876***	1.798***	1.676**
	(0.0097)	(0.0030)	(0.0321)	(0.0091)	(0.6820)	(0.8170)
Province Fe	N	Y	N	Y	Y	Y
Year Fe	N	N	Y	Y	N	Y
Observations	390	390	390	390	390	390
R^2^	0.037	0.012	0.051	0.07	0.029	0.086

Note: *, ** and *** indicate statistical significance at the level of 10%, 5% and 1%, respectively; Standard errors are reported in parentheses.

Among the control variables, at a level of 5%, the influence coefficient of government health spending (*Gov*_*it*_) on the allocation efficiency of medical resource is markedly negative. The market mechanism is seen as an efficient way to increase efficiency in the distribution of medical and health resources, while the government’s involvement is more focused on ensuring the equity of access to medical and health resources [47]. The rise in government spending on medical and healthcare may displace spending on public and social health, resulting in an insufficient investment in total health spending and impeding the improvement of MRAE.

### Parallel Trend Test and Dynamic Effects Analysis

**FIGURE 2:**
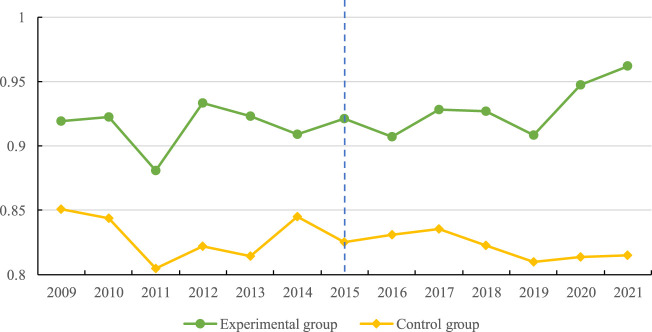
Trends of healthcare resource efficiency in pilot and non-pilot provinces during 2009–2021. (China, 2009–2021).

This study further examines the trend of MRAE between pilot provinces and non-pilot provinces in the period 2009–2021. Figure 2 shows that between 2009 and 2015, there is no significant difference between pilot provinces and non-pilot provinces. After the implementation of the comprehensive medical reform pilot strategy, the changing trend of MRAE in the pilot and non-pilot provinces largely varies, supporting the parallel trend theory.

**FIGURE 3:**
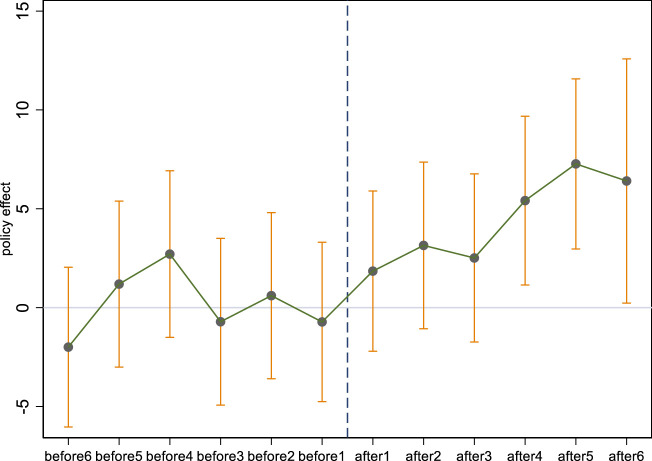
Changes in the estimated coefficients of the comprehensive medical reform pilot policy. (China, 2009–2021).

Additionally, the event analysis approach is also conducted to test the parallel trend and analyze the dynamic influence of comprehensive medical reform pilot strategy on MRAE. Supplementary File S5 reports the test results without and with control variables respectively. We can see that du*beforei (i = 1, 2, 3,..., 6) failed to pass the significance test, this shows that there is no significant difference in the efficiency of medical resource allocation between the treatment group and the control group before the implementation of the pilot policy of comprehensive medical reform. Thus, the comprehensive medical reform pilot policy accepts the parallel trend hypothesis, which confirms the validity of the previous estimation results. In the dynamic effect of the pilot comprehensive medical reform, the estimated coefficient of du*after_*i*_ (*i* = 1, 2, 3) is not significant, and the estimated coefficient of du*after_*i*_ (*i* = 4, 5, 6) is significantly positive at the level of 10%. It shows that the pilot policy of comprehensive medical reform has a certain time lag and has a continuous effect on the improvement of efficiency of medical resource allocation. Figure 3 shows the changing trend of the regression estimated coefficients of the comprehensive medical reform pilot policy year by year, which again confirms the estimated results of the parallel trend and dynamic effect test above.

### Placebo Test

It’s possible that the policy impact of the comprehensive medical reform pilot program has nothing to do with the increased efficiency of medical resource allocation. Instead, it is affected by uncontrollable or additional laws. In order to exclude the influence of other random factors, referring to the previous research [48, 49], a placebo test was carried out after the aforementioned method had been carried out 1,000 times. The estimated coefficients of du*dt kernel density distribution are shown in crystal clear detail in Supplementary File S6. The estimated coefficients for the placebo test differ significantly from the actual ones, and the majority of *p* values are higher than 0.1. Therefore, it is established from a counterfactual standpoint that the pilot program for comprehensive medical reform can increase the allocation efficiency of medical resources. The other robustness analysis can be seen in Supplementary File S10.

### Heterogeneity Analysis

#### Geographical Position

Based on the geographical location, this paper firstly analyzes the impact of the pilot policy of comprehensive medical reform on the efficiency of medical resource allocation in East China, Central China, and West China, respectively (see Supplementary File S7). It can be seen that the impact of the CMRP on the efficiency of medical resource allocation is significantly positive.in the East China and Central China, at the level of 10%. This shows that the efficiency of medical resource allocation in Shanghai, Beijing, Shanxi and other pilot provinces of medical reform in the eastern and central regions has been significantly improved after the policy impact. However, in western region, the coefficient of du*dt is positive, but fails to pass the significance test. West China has limited medical resources *per capita*. It is difficult to take the lead in medical and healthcare reform legislation because of the growing gap between the demand and the available resources.

#### Efficiency of Medical Resource Allocation

The entire sample is divided into high and low groups in this study based on the median of efficiency of medical resource allocation (0.959). According to Supplementary File S8, the comprehensive medical reform pilot policy’s influence effect is significantly positive in the high efficiency group while it fails to pass the significance test in the low efficiency group. It indicates that the level of medical services has increased as a result of the pilot comprehensive medical reform, which has optimized the allocation of medical and health resources in those pilot provinces with high MRAE efficiency.

#### Population Size

Supplementary File S9 reports the heterogeneity analysis of population size. It can be found that the policy impact of the pilot comprehensive medical reform is not significant in the provinces with populations below the median (3,894.49). In the provinces with populations above the median, the coefficient of du*dt is significantly positive at the 1% confidence level. The need for the improvement of medical resources allocation efficiency is likely higher in provinces with a larger population, where the policy effect of pilot comprehensive medical reform can be more effective.

## Discussion

The comprehensive medical reform pilot strategy is a significant effort to improve medical resource capability for the circumstance in China. This study is based on panel data from 30 Chinese provinces and cities from 2009 to 2021. The allocation efficiency of medical resources in each province is assessed using the SBM-DDF model under meta-frontier, which takes into account undesirable output. The policy impact of the comprehensive medical reform pilot program on efficiency of medical resource allocation is investigated using the multi-period DID method.

The findings are as follows: 1) China’s medical resource allocation efficiency fluctuated during the sample period. East China has a higher efficiency value than Central and West China, which is in line with study by Sun and Luo [3]. Eastern China has a concentration of strong medical resources. Additionally, the advancement of technology has ensured medical reform. 2) The comprehensive medical reform pilot strategy has greatly increased the China’s medical resource allocation efficiency. And, the promotion effect remains valid after a series of robustness tests, which includes changing the regression technique, lowering the sample size, addressing the potential endogeneity issues, doing a placebo test. Dynamic effect analysis show that the promotion effect of the comprehensive medical reform pilot program is substantial. 3) Heterogeneity analysis demonstrates that only East China and Central China, as well as the groups with higher medical and health resource efficiency, and larger population scale, have considerably favorable effects from the pilot comprehensive medical reform. Relatively old medical facilities and working conditions in West China make it difficult to attract medical workers. Based on the above findings, the policy suggestions are proposed as follows.

First off, there is still a lot of potential for improvement of medical resources allocation efficiency in the central and western regions. To facilitate coordinated regional development of medical resource allocation capability, the government should concentrate on developing a regional cooperation framework. In order to strengthen the ability of the central and western regions to allocate medical resources, it is crucial to maximize the leadership role of the eastern provinces.

Second, the pilot program for comprehensive medical reform has had a significant impact on improving the medical resources allocation efficiency. Therefore, as the medical and health system reform process moves forward, the advanced experience of the pilot provinces should be improved upon and encouraged. The government should keep enlarging the pilot provinces' purview and encourage the thorough development of a complete medical reform to increase the efficiency of allocating medical resources.

Thirdly, medical resource allocation should be optimized by the market. A major contributing factor to the mismatch between the supply and demand of medical resources is market failure. Creating an effective market is critical to removing resource barriers across healthcare facilities and achieving the goal of rational resource allocation.
