# Supplementary material for: Can Comprehensive Medical Reform Improve the Efficiency of Medical Resource Allocation? Evidence From China
Source: Int J Public Health. 2023 Dec 21;68:1606602. doi: 10.3389/ijph.2023.1606602 (PMC10764414; doi:10.3389/ijph.2023.1606602)
Supplement: Supplementary file 1 [file DataSheet4.docx]

Multicollinearity analysis. (China, 2009-2021)

|  | MRAE_it_ | du*dt | lnPGDP_it_ | lnPOP_it_ | Gov_it_ | Idu_it_ | lnPat_it_ |
| --- | --- | --- | --- | --- | --- | --- | --- |
| MRAE_it_ | 1.000 |  |  |  |  |  |  |
| du*dt | 0.193*** | 1.000 |  |  |  |  |  |
| lnPGDP_it_ | 0.207*** | 0.398*** | 1.000 |  |  |  |  |
| lnPOP_it_ | -0.020 | -0.028 | 0.055 | 1.000 |  |  |  |
| Gov_it_ | 0.223*** | 0.198*** | 0.060 | 0.384*** | 1.000 |  |  |
| Idu_it_ | 0.162*** | 0.040 | 0.532*** | -0.248*** | 0.040 | 1.000 |  |
| lnPat_it_ | 0.177*** | 0.279*** | 0.708*** | 0.635*** | 0.260*** | 0.276*** | 1.000 |
| VIF |  | 1.42 | 4.46 | 4.69 | 1.32 | 1.89 | 7.60 |
| 1/VIF |  | 0.704 | 0.224 | 0.213 | 0.757 | 0.53 | 0.132 |

Note: *, ** and *** indicate statistical significance at the level of 10%, 5% and 1%, respectively.
