# Supplementary material for: Can Comprehensive Medical Reform Improve the Efficiency of Medical Resource Allocation? Evidence From China
Source: Int J Public Health. 2023 Dec 21;68:1606602. doi: 10.3389/ijph.2023.1606602 (PMC10764414; doi:10.3389/ijph.2023.1606602)
Supplement: Supplementary file 2 [file DataSheet7.docx]

Heterogeneity analysis of geographical position. (China, 2009-2021)

|  | East China | Central China | West China |
| --- | --- | --- | --- |
| du*dt | 0.0217* | 0.0666** | 0.0118 |
|  | (0.0116) | (0.0333) | (0.0158) |
| Constant | 1.4120 | -0.1050 | 3.108* |
|  | (1.0490) | (2.4120) | (1.6860) |
| Controls | Y | Y | Y |
| Province Fe | Y | Y | Y |
| Year Fe | Y | Y | Y |
| Observations | 143 | 104 | 143 |
| R^2^ | 0.207 | 0.319 | 0.234 |

Note: *, ** and *** indicate statistical significance at the level of 10%, 5% and 1%, respectively; Standard errors are reported in parentheses.
