# Supplementary material for: Can Comprehensive Medical Reform Improve the Efficiency of Medical Resource Allocation? Evidence From China
Source: Int J Public Health. 2023 Dec 21;68:1606602. doi: 10.3389/ijph.2023.1606602 (PMC10764414; doi:10.3389/ijph.2023.1606602)
Supplement: Supplementary file 4 [file DataSheet3.docx]

Changing trend of China's healthcare resource efficiency and technical gap ratio index under group frontier and meta-frontier from 2009 to 2021. (China, 2009-2021)
