# Supplementary material for: Can Comprehensive Medical Reform Improve the Efficiency of Medical Resource Allocation? Evidence From China
Source: Int J Public Health. 2023 Dec 21;68:1606602. doi: 10.3389/ijph.2023.1606602 (PMC10764414; doi:10.3389/ijph.2023.1606602)
Supplement: Supplementary file 5 [file DataSheet9.docx]

Population size heterogeneity analysis. (China, 2009-2021)

|  | High population group | Low population group |
| --- | --- | --- |
| du*dt | 0.0476** | 0.0065 |
|  | (0.0189) | (0.0133) |
| Constant | 2.5690 | 1.450** |
|  | (2.3290) | (0.7060) |
| Controls | Y | Y |
| Province Fe | Y | Y |
| Year Fe | Y | Y |
| Observations | 195 | 195 |
| R^2^ | 0.117 | 0.135 |

Note: *, ** and *** indicate statistical significance at the level of 10%, 5% and 1%, respectively; Standard errors of the estimated coefficients are reported in parentheses.
