# Supplementary material for: Can Comprehensive Medical Reform Improve the Efficiency of Medical Resource Allocation? Evidence From China
Source: Int J Public Health. 2023 Dec 21;68:1606602. doi: 10.3389/ijph.2023.1606602 (PMC10764414; doi:10.3389/ijph.2023.1606602)
Supplement: Supplementary file 6 [file DataSheet5.docx]

Test of parallel trends and dynamic effects. (China, 2009-2021)

| No control variables | | | |  | Add control variables | | | |
| --- | --- | --- | --- | --- | --- | --- | --- | --- |
| du*before 6 | -0.0195 | du*after 4 | 0.0476** |  | du*before 6 | -0.0199 | du*after 4 | 0.0542** |
|  | (0.0205) |  | (0.0215) |  |  | (0.0205) |  | (0.0217) |
| du*before 5 | 0.0144 | du*after 5 | 0.0653*** |  | du*before 5 | 0.0119 | du*after 5 | 0.0727*** |
|  | (0.0214) |  | (0.0216) |  |  | (0.0213) |  | (0.0219) |
| du*before 4 | 0.0282 | du*after 6 | 0.0519* |  | du*before 4 | 0.0271 | du*after 6 | 0.0641** |
|  | (0.0215) |  | (0.0311) |  |  | (0.0214) |  | (0.0314) |
| du*before 3 | -0.0083 |  |  |  | du*before 3 | -0.0071 | lnPGDPit | -0.0225 |
|  | (0.0215) |  |  |  |  | (0.0214) |  | (0.0445) |
| du*before 2 | 0.0048 |  |  |  | du*before 2 | 0.0061 | lnPOPit | -0.1040 |
|  | (0.0214) |  |  |  |  | (0.0213) |  | (0.0836) |
| du*before 1 | -0.0077 |  |  |  | du*before 1 | -0.0072 | Gov_it_ | -0.966** |
|  | (0.0205) |  |  |  |  | (0.0205) |  | (0.4890) |
| du*after 1 | 0.0173 |  |  |  | du*after 1 | 0.0185 | Idu_it_ | 0.0074 |
|  | (0.0205) |  |  |  |  | (0.0206) |  | (0.0172) |
| du*after 2 | 0.0267 |  |  |  | du*after 2 | 0.0315 | lnPat_it_ | 0.0060 |
|  | (0.0214) |  |  |  |  | (0.0214) |  | (0.0109) |
| du*after 3 | 0.0177 | Constant | 0.879*** |  | du*after 3 | 0.0251 | Constant | 1.960** |
|  | (0.0215) |  | (0.0094) |  |  | (0.0216) |  | (0.8130) |
| Province Fe | Y | Year Fe | Y |  | Province Fe | Y | Year Fe | Y |
| Observations | 390 | R^2^ | 0.111 |  | Observations | 390 | R^2^ | 0.131 |

Note: *, ** and *** indicate statistical significance at the level of 10%, 5% and 1%, respectively; Standard errors are reported in parentheses.
