# Supplementary material for: Can Comprehensive Medical Reform Improve the Efficiency of Medical Resource Allocation? Evidence From China
Source: Int J Public Health. 2023 Dec 21;68:1606602. doi: 10.3389/ijph.2023.1606602 (PMC10764414; doi:10.3389/ijph.2023.1606602)
Supplement: Supplementary file 7 [file DataSheet10.docx]

**SBM-DDF model**

It is assumed that a total of *m* factors of production are invested in each province（*X_M_*，*i*=1, 2,…,*m*），get *R* desired outputs（*Y_R_*, *i*=1, 2,…,*r*） and V undesirable outputs（*B_V_*, *i*=1, 2,…,*r*）. Then the set of production possibilities under group frontier is as follows:

$P^{group}=\left\{\left(x^{h},y^{h},b^{h}\right):\sum_{n=1}^{N_{h}} \xi_{n}^{h}x_{n}^{h}\leq x^{h},\sum_{n=1}^{N_{h}} \xi_{n}^{h}y_{n}^{h}\geq y^{h},\sum_{n=1}^{N_{h}} \xi_{n}^{h}b_{n}^{h}\leq b^{h}\right\}$ （1）

The measurement model of China's medical resource allocation efficiency under group frontier can be defined as follows:

$\begin{matrix} \overrightarrow{S_{V}^{group}}\left(x,y,b,g^{x},g^{y},g^{b}\right)\text{=max}\frac{\frac{1}{M}\sum_{m=1}^{M} \frac{s_{mo}^{x}}{g_{m}^{x}}+\frac{1}{R+V}\left(\sum_{r=1}^{R} \frac{s_{ro}^{y}}{g_{r}^{y}}+\sum_{v=1}^{V} \frac{s_{vo}^{b}}{g_{v}^{b}}\right)}{2} \\ s.t.\begin{matrix} & \end{matrix}\sum_{n=1}^{N} \lambda_{n}^{h}x_{mn}=x_{mo}-s_{mo}^{x}; \\ \begin{matrix} & & \end{matrix}\sum_{n=1}^{N} \lambda_{n}^{h}y_{rn}=y_{ro}+s_{ro}^{y}; \\ \begin{matrix} & & \end{matrix}\sum_{n=1}^{N} \lambda_{n}^{h}b_{vn}=b_{vo}-s_{vo}^{b}; \\ \begin{matrix} & & \end{matrix}s_{mo}^{x}>0;s_{ro}^{y}>0;s_{vo}^{b}>0;\sum\lambda_{n}^{h}\text{=1；} \\ \begin{matrix} & & \end{matrix}m=1,2,\cdots,M;r=1,2,\cdots,R;v=1,2,\cdots,V. \end{matrix}$ （2）

Considering the heterogeneity of production technology, this paper continues to divide N DMUs into *h* groups and constructs the production technology set *P^meta^* as follows:

$P^{meta}=\left\{\left(x,y,b\right):\sum_{h=1}^{H} \sum_{n=1}^{N_{h}} \lambda_{n}^{h}x_{n}^{h}\leq x^{h},\sum_{h}^{H} \sum_{n=1}^{N_{h}} \lambda_{n}^{h}y_{n}^{h}\geq y^{h},\sum_{h}^{H} \sum_{n=1}^{N_{h}} \lambda_{n}^{h}b_{n}^{h}\leq b^{h},\lambda_{n}^{h}\geq0,n=1,2,\cdots,N_{h}\right\}$ （3）

Of which，$P^{meta}=P^{1}\cup P^{2}\cup\cdots\cup P^{H}$。

The SBM-DDF model under meta-frontier is shown in Formula (4) :

$\begin{matrix} \overrightarrow{S_{V}^{meta}}\left(x,y,b,g^{x},g^{y},g^{b}\right)\text{=max}\frac{\frac{1}{M}\sum_{m=1}^{M} \frac{s_{mko}^{x}}{g_{m}^{x}}+\frac{1}{R+V}\left(\sum_{r=1}^{R} \frac{s_{rko}^{y}}{g_{r}^{y}}+\sum_{v=1}^{V} \frac{s_{vko}^{b}}{g_{v}^{b}}\right)}{2} \\ s.t.\begin{matrix} & \end{matrix}\sum_{h=1}^{H} \sum_{n=1}^{N_{h}} \lambda_{n}^{h}x_{mhn}=x_{mko}-s_{mko}^{x}; \\ \begin{matrix} & & \end{matrix}\sum_{h=1}^{H} \sum_{n=1}^{N_{h}} \lambda_{n}^{h}y_{rhn}=y_{rko}+s_{rko}^{y}; \\ \begin{matrix} & & \end{matrix}\sum_{h=1}^{H} \sum_{n=1}^{N_{h}} \lambda_{n}^{h}b_{vhn}=b_{vko}-s_{vko}^{b}; \\ \begin{matrix} & & \end{matrix}0<s_{mko}^{x}\leq1;0<s_{rko}^{y}\leq1;0<s_{vko}^{b}\leq1;\sum\lambda_{n}^{h}=1; \\ \begin{matrix} & & \end{matrix}m=1,2,\cdots,M;r=1,2,\cdots,R;v=1,2,\cdots,V. \end{matrix}$ （4）

Of which，$\overrightarrow{S_{V}^{group}}$和$\overrightarrow{S_{V}^{meta}}$ represent the SBM-DDF under group frontier and meta-frontier, respectively; *x、y* and *b* are the values of inputs, desired outputs, and undesired outputs, respectively; *s^x^*、*s^y^* and *s^b^* are slack variables that represent the amount of input redundancy, desired output shortfall, and undesired output surplus; *g^x^*、*g^y^* and *g^b^* denote the direction vectors of input reduction, desired output increase, and undesired output decrease, respectively. Based on the theory of health production proposed by Grossman [1], the production technology of medical resources has variable returns to scale. Therefore, this paper is selected $\sum\lambda_{n}^{h}=1$.

Furthermore, following the study of Luo et al. [33], we further calculate the technology gap ratio, which measures the difference between the meta-frontier and the group frontier technology level. It can reflect China’s MRAE improvement potential. The specific calculating formula is built as follows.

$TGR=\frac{\overrightarrow{S_{V}^{meta}}}{\overrightarrow{S_{V}^{group}}}\in\left[0,1\right]$ （5）

**Robustness analysis**

To ensure the robustness of the prior estimation results, we further conduct robustness analysis by substituting the estimate method, shortening the sample duration, minimizing endogeneity. Tables 7 display the findings.

(1) Modifying the estimating technique. Considering the allocation efficiency value of medical resources has a truncated distribution, the results based on OLS might be biased. Thus, the relationship between the two is further re-estimated using Tobit regression. It can be found that the coefficient of *du*dt* is 0.0306, which has a 1% level of statistical significance. This demonstrates that comprehensive medical reform pilot strategy has increased the allocation efficiency of medical resources in the pilot provinces, and verifies the reliability of the previous results.

(2) Modifying the sample size. The Column (2) in Table 1 shows that the estimated coefficient of *du*dt* is still considerably positive at the confidence level of 10% with the samples from 2014 to 2021, which is consistent with the earlier findings.

(3) Endogeneity analysis. To mitigate the potential bias caused by endogeneity issues on the estimation results, this study refers to the work of Li et al. [2] and performs regression by introducing one-period-lagged terms of the dependent variable and one-period-lagged terms of all control variables, respectively. The results are reported in Column (3) and (4) in Table 7. It can be found that the primary explanatory variable *du*dt* coefficient sign agrees with the benchmark regression results, supporting the prior assessment of the impact of the comprehensive medical reform pilot strategy.

**TABLE 1|** Robust analysis. (China, 2009-2021)

|  | (1) | (2) | (3) | (4) |
| --- | --- | --- | --- | --- |
|  | Tobit | 2014-2021 | Control initial efficiency | lagged variables |
| du*dt | 0.0306*** | 0.0295* | 0.0189* | 0.0228** |
|  | (0.0101) | (0.0160) | (0.0108) | (0.0111) |
| Constant | 1.757** | (2.4950) | 0.1360 | 0.8150 |
|  | (0.7660) | -1.5880 | (0.8820) | (0.8880) |
| Controls | Y | Y | Y | Y |
| Province Fe | Y | Y | Y | Y |
| Year Fe | Y | Y | Y | Y |
| Observations | 390 | 330 | 360 | 360 |
| Log likelihood/R^2^ | 643.38335 | 0.101 | 0.134 | 0.069 |

Note: *, ** and *** indicate statistical significance at the level of 10%, 5% and 1%, respectively; Standard errors are reported in parentheses.

Reference

1. Grossman, M., 1972. On the concept of health capital and the demand for health. Journal of Political Economy,80(2):223-55.
2. Li P, Lu Y, Wang J. Does flattening government improve economic performance? Evidence from China. Journal and Development Economics, 2016, 123: 18-37.
