# Supplementary material for: Can Comprehensive Medical Reform Improve the Efficiency of Medical Resource Allocation? Evidence From China
Source: Int J Public Health. 2023 Dec 21;68:1606602. doi: 10.3389/ijph.2023.1606602 (PMC10764414; doi:10.3389/ijph.2023.1606602)
Supplement: Supplementary file 9 [file DataSheet8.docx]

Heterogeneity analysis of efficiency of medical resource allocation. (China, 2009-2021)

|  | High efficiency group | Low efficiency group |
| --- | --- | --- |
| du*dt | 0.0035** | 0.0230 |
|  | (0.0017) | (0.0168) |
| Constant | 1.354*** | 2.795** |
|  | (0.2270) | (1.1040) |
| Controls | Y | Y |
| Province Fe | Y | Y |
| Year Fe | Y | Y |
| Observations | 195 | 195 |
| R^2^ | 0.108 | 0.175 |

Note: *, ** and *** indicate statistical significance at the level of 10%, 5% and 1%, respectively; Standard errors are reported in parentheses.
