# Supplementary material for: Can Comprehensive Medical Reform Improve the Efficiency of Medical Resource Allocation? Evidence From China
Source: Int J Public Health. 2023 Dec 21;68:1606602. doi: 10.3389/ijph.2023.1606602 (PMC10764414; doi:10.3389/ijph.2023.1606602)
Supplement: Supplementary file 10 [file DataSheet1.docx]

Correlation and descriptive analysis of input-output variables. (China, 2009-2021)

|  |  | HE | HP | MIB | MI | OV | DR | BU | MOR | EM |
| --- | --- | --- | --- | --- | --- | --- | --- | --- | --- | --- |
| Correlation analysis | HE | 1.000 |  |  |  |  |  |  |  |  |
|  | HP | 0.863*** | 1.000 |  |  |  |  |  |  |  |
|  | MIB | 0.831*** | 0.967*** | 1.000 |  |  |  |  |  |  |
|  | MI | 0.508*** | 0.812*** | 0.816*** | 1.000 |  |  |  |  |  |
|  | OV | 0.812*** | 0.926*** | 0.845*** | 0.682*** | 1.000 |  |  |  |  |
|  | DR | -0.093* | -0.098* | -0.128** | -0.110** | -0.042 | 1.000 |  |  |  |
|  | BU | -0.090* | 0.080 | 0.061 | 0.128** | 0.248*** | -0.052 | 1.000 |  |  |
|  | MOR | 0.095* | -0.083 | -0.096* | -0.195*** | -0.016 | 0.013 | 0.109** | 1.000 |  |
|  | EM | -0.178*** | -0.155*** | -0.154*** | 0.014 | -0.252*** | 0.026 | -0.322*** | 0.190*** | 1.000 |
| Descriptive analysis | Mean | 1464.530 | 357832.860 | 232882.310 | 32414.480 | 24686.090 | 1.000 | 83.400 | 0.150 | 0.100 |
|  | Std. Dev | 1174.210 | 224570.360 | 151390.130 | 22041.030 | 18690.100 | 0.010 | 7.940 | 0.340 | 0.060 |
|  | Min | 77.990 | 34429.000 | 19223.000 | 4129.000 | 1784.020 | 0.950 | 48.300 | 0.000 | 0.020 |
|  | Max | 8249.260 | 1058702.000 | 721329.000 | 88162.000 | 89179.770 | 1.040 | 100.200 | 2.910 | 0.400 |
